# Genomisches Neugeborenenscreening – Forschungsansätze, Herausforderungen und Chancen

**DOI:** 10.1007/s00103-023-03777-2

**Published:** 2023-10-13

**Authors:** Heiko Brennenstuhl, Christian P. Schaaf

**Affiliations:** Institut für Humangenetik, Universität Heidelberg, Heidelberg, Baden-Württemberg Deutschland

**Keywords:** Seltene Erkrankungen, Angeborene Erkrankungen, Whole-Genome Sequencing, Populations-Screening, Rare diseases, Congenital diseases, Whole-genome sequencing, Population screening

## Abstract

Die Anwendung von Hochdurchsatz-Sequenziermethoden für ein populationsbasiertes genomisches Neugeborenenscreening (gNBS) bietet zahlreiche Chancen für die Verbesserung der Bevölkerungsgesundheit. Ein solches würde ermöglichen, die Diagnose zahlreicher genetischer Erkrankungen bereits in einem frühen, präsymptomatischen Stadium zu stellen, und böte große Flexibilität bei der Auswahl und Erweiterung von Zielkrankheiten. National und international werden daher Anstrengungen unternommen, um die ethischen, rechtlichen, sozialen, psychologischen und technischen Aspekte des gNBS zu untersuchen. Neben den vielen Chancen existieren auch zahlreiche Herausforderungen und noch offene Fragen: Wann und wie sollten Erziehungsberechtigte über ein solches Screening informiert werden? Auf welche Krankheiten sollte gescreent werden? Wie soll mit Zufallsbefunden oder der Feststellung einer genetischen Veranlagung umgegangen werden? Sollen die Daten langfristig gespeichert werden und, wenn ja, wie kann dies sicher geschehen? Unter der Voraussetzung einer angemessenen Rechtsgrundlage und eines transparenten Einwilligungsprozesses hat das genomische Neugeborenenscreening das Potenzial, die Art und Weise, wie wir angeborene Krankheiten diagnostizieren, grundlegend zu verändern. Es gibt jedoch noch viel zu tun. Um ein gutes Verständnis und eine ausreichende Akzeptanz des gNBS bei allen Beteiligten zu erreichen und so den Nutzen für die Bevölkerung zu maximieren, ist ein öffentlicher Diskurs über die Möglichkeiten und Grenzen des gNBS von zentraler Bedeutung. Dieser Beitrag hat das Ziel, einen Überblick über die innovativen technischen Entwicklungen in der Humangenetik, nationale und internationale Forschungsansätze sowie über Chancen und Herausforderungen bei der Entwicklung eines genomischen Neugeborenenscreenings zu geben.

## Abstract (en)

The application of high-throughput sequencing methods for population-based genomic newborn screening offers numerous opportunities for improving population health. The use of genome-based sequencing technology holds potential to enable the diagnosis of virtually any genetic disorder at an early stage and offers great flexibility when it comes to selection and expansion of target diseases. National and international efforts are therefore being made to investigate the ethical, legal, social, psychological, and technical aspects of genomic newborn screening. In addition to the many opportunities, there are numerous challenges and questions that remain to be answered: When and how should legal guardians be informed about such screening? Which diseases should be screened for? How should incidental findings or identification of a genetic predisposition be dealt with? Should data be stored long term and if so, how can this be done securely? Provided there is an appropriate regulatory framework and a transparent consent process, genomic newborn screening has the potential to fundamentally change the way in which we screen for congenital diseases. However, there is still much to be done. To achieve understanding and acceptance of genomic newborn screening amongst all stakeholders and thus to maximize its benefits for the population, a public discourse on the possibilities and limitations of genomic newborn screening is of critical importance. This article aims to provide an overview of the innovative technical developments in the field of human genetics, describe national and international approaches, and discuss challenges and opportunities of genomic newborn screening development.

## Einleitung

Das Neugeborenenscreening (*Newborn Screening* – NBS) stellt eine der effektivsten bevölkerungsmedizinischen Maßnahmen der Sekundärprävention dar und hat die vollständige Erkennung und qualitätsgesicherte Therapie von Neugeborenen mit angeborenen und behandelbaren Erkrankungen zum Ziel [1–4]. Derzeit wird das Blut Neugeborener im erweiterten Neugeborenenscreening in Deutschland auf 13 Stoffwechselerkrankungen, 2 Endokrinopathien sowie die Mukoviszidose, schwere kombinierte Immundefekte (SCID) und andere T‑Zell-Defizienzen, die Sichelzellanämie und seit Oktober 2021 auch auf die spinale Muskelatrophie (SMA) untersucht. Die Analytik im Rahmen des NBS fällt unter § 3, Nr. 9 des Gendiagnostikgesetzes (GenDG, „Genetische Reihenuntersuchungen“; [5]). Grundlage für das Neugeborenenscreening ist die vom Gemeinsamen Bundesausschuss (G-BA) gemäß § 26 SGB V beschlossene „Kinder-Richtlinie“ zur Früherkennung von Krankheiten, die seit ihrem Inkrafttreten bereits mehrfach, zuletzt am 21.04.2022, überarbeitet wurde [6].

Die Mehrheit der aktuell gescreenten Erkrankungen wird anhand eines auffälligen biochemischen Profils diagnostiziert. Voraussetzung hierfür ist die Verfügbarkeit eines oder mehrerer biochemischer Metaboliten, welche bei Erkrankung im Blut der Patienten in auffälliger Konzentration vorliegen und mit der Messmethodik sicher detektiert werden können [7]. Molekulargenetische Methoden zur Identifizierung einer kausalen genetischen Erkrankungsursache werden bislang vorwiegend bei Folgeuntersuchungen im Rahmen der Konfirmationsdiagnostik (als sog. Second-tier-Verfahren) angewendet. Mit der Untersuchung auf SMA wurde 2021 erstmals eine molekulargenetische Methode Bestandteil des Screening-Prozesses, in dem mittels quantitativer Polymerase-Kettenreaktion die aus Trockenblutkarten extrahierte DNA auf das Vorliegen einer homozygoten Deletion von Exon 7 des *SMN1*-Gens untersucht wird [8, 9].

Die seit den späten 1990er-Jahren verfügbare (Elektrospray-Ionisierungs‑)Tandem-Massenspektrometrie (MS/MS) zur Messung biochemischer Metaboliten hat unsere Fähigkeit, Krankheiten bei asymptomatischen Neugeborenen zu erkennen, erheblich verbessert [10, 11]. Nichtsdestotrotz wurde ihre Verwendung, besonders in den Anfangsjahren, kritisch diskutiert [12]. Methoden der Hochdurchsatz-Sequenzierung menschlicher DNA könnten einen kosteneffektiven Screening-Ansatz für zahlreiche weitere unmittelbar behandelbare Erkrankungen des Kindesalters darstellen, insbesondere wenn keine biochemischen Marker im klassischen Sinne verfügbar sind. In diesem Artikel legen wir die innovativen technischen Entwicklungen im Feld der Humangenetik dar, stellen nationale und internationale Forschungsansätze für ein genomisches Neugeborenenscreening (gNBS) vor und diskutieren die damit assoziierten Chancen und Herausforderungen.

## Entwicklungen der genetischen Diagnostik

**Figure 1:**
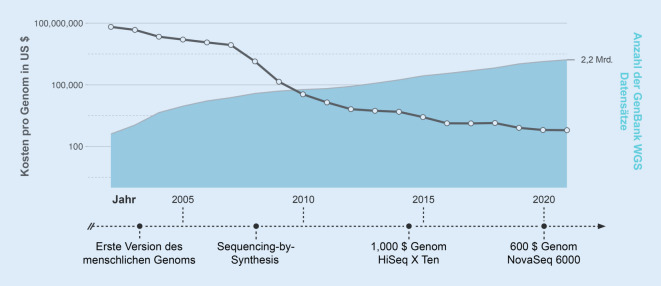

Kaum ein Feld der biomedizinischen Forschung und klinischen Diagnostik hat in den letzten Jahren so stark von technischer Innovation und Fortschritt profitiert wie die Humangenetik. Nach der Entdeckung der Doppelhelixstruktur der DNA durch Watson und Crick im Jahr 1953 verging ein halbes Jahrhundert, bis 2001 die Sequenz des Euchromatin-Anteils der menschlichen DNA durch das *Human Genome Project* veröffentlicht wurde [13–15]. Für die Erstbeschreibung waren 13 Jahre, knapp 2,7 Mrd. US-Dollar und ein Team von mehr als 1000 internationalen Wissenschaftlern notwendig, die mittels klassischer Sanger-Sequenzierung die 3 Mrd. Basen des menschlichen Genoms entschlüsselten. Die technische Revolution des „massively parallel sequencing“, auch *Next-Generation-Sequencing* (NGS) genannt, ermöglichte nur wenige Jahre später, das Genom eines einzelnen Menschen in einem Zeitraum von lediglich 2 Monaten zu einem Bruchteil der Kosten zu sequenzieren [16]. Seither kam es zu einem stetigen Zuwachs der Leistungsfähigkeit moderner Sequenziergeräte und einem konsekutiven Rückgang der Sequenzierkosten, welche mittlerweile unter 1000 US-Dollar pro Genom liegen (Abb. 1; [17]). Die Zahl der Genomsequenzen in GenBank, einer öffentlichen DNA-Datenbank, hat sich 2002–2021 ca. alle 18 Monate verdoppelt. Aktuelle Entwicklungen beinhalten sogenannte Long-read-Sequenzierverfahren wie das *PacBio Circular Consensus Sequencing (HiFi) *oder das *Oxford Nanopore Ultralong-Read Sequencing*, welche derzeit noch vorwiegend im Forschungskontext genutzt werden [18, 19]. Möglicherweise werden auch diese Techniken nach und nach den Einzug in die klinische Versorgung finden.

Beim NGS werden in einem skalierbaren Untersuchungsansatz mehrere Millionen DNA-Fragmente parallel sequenziert. Dies ermöglicht eine Hochdurchsatzanalytik und damit eine drastische Reduktion der assoziierten Kosten, während die Qualität vergleichbar zur klassischen Sanger-Sequenzierung bleibt. Bei einer Genpanel-Analyse oder beim Whole-Exome-Sequencing (WES) wird vor der Sequenzierung eine sogenannte Library erstellt, in der bestimmte oder aber die Gesamtheit der proteinkodierenden Bereiche der DNA mittels Polymerase-Kettenreaktion („polymerase chain reaction“, PCR) angereichert und an sogenannte Adapter gekoppelt werden. Beim Whole-Genome-Sequencing (WGS) kann die Erstellung der Library durch einen möglichen Verzicht auf die PCR deutlich beschleunigt werden. Die DNA-gekoppelten Adapter ermöglichen die Bindung des DNA-Templates an speziell beschichtete Oberflächen sogenannter Flow Cells. Auf der Oberfläche einer solchen Flow Cell kommt es nachfolgend zur klonalen Amplifizierung eines Clusters, der lokalen Vermehrung dieses DNA-Templates. Die Sequenzierung erfolgt dann schrittweise durch das Binden komplementärer, fluoreszierender Nukleotide. Nach jeder Bindung wird das Cluster von einer Lichtquelle angeregt und ein charakteristisches Fluoreszenzsignal emittiert. Aus der Wellenlänge der Emission und der Signalintensität kann ermittelt werden, welches Nukleotid eingebaut wurde. Diese Technologie wird als *Sequencing-by-Synthesis* bezeichnet und ermöglicht die parallele Sequenzierung von Millionen DNA-Fragmenten.

Aus WES/WGS-Analysen resultieren enorme Datenmengen, welche eine bioinformatische Verarbeitung notwendig machen. In einem ersten Schritt (sog. Alignment) werden die ermittelten und häufig nur sehr kurzen DNA-Fragmente überlappend aneinandergelegt und am Referenzgenom (bspw. GRCh38, *Genome Research Consortium Human Build 38, *auch hg38 genannt) ausgerichtet. In diesem Schritt wird auch die Sequenziertiefe ermittelt, die angibt, wie häufig eine eindeutige Base an der jeweiligen Stelle in der rekonstruierten Sequenz identifiziert wurde (sog. Coverage). Eine Coverage von ≥ 30 wird als Voraussetzung für die zuverlässige Interpretierbarkeit der genetischen Sequenz betrachtet. Im nächsten Schritt werden die komplexen Rohdaten reduziert, indem nur relevante Abweichungen vom Referenzgenom selektiert, annotiert und als separate Datei gespeichert werden. Die Interpretation der relevanten Abweichungen vom Referenzgenom erfolgt dann anhand der *American-College-of-Medical-Genetics-and-Genomics-*(ACMG-)Kriterien, welche helfen, die Pathogenität genetischer Varianten zu klassifizieren, um so die klinische Entscheidungsfindung zu unterstützen [20]. Bewertet werden hierbei unter anderem Hinweise auf einen kausalen Zusammenhang zwischen einer genetischen Variante und einer Krankheit, die Position im Gen, die Häufigkeit, das Vorkommen in Kontrollpopulationen und funktionelle sowie computergestützte Belege für oder gegen die Pathogenität der jeweiligen Variante. Mittlerweile werden NGS-Verfahren flächendeckend in der molekulargenetischen Diagnostik verwendet. Vor- und Nachteile der am häufigsten verwendeten Methoden sind in Tab. 1 dargestellt.

**Table 1:** 

	Genpanel-Analyse	Whole-Exome-Sequencing (WES)	Whole-Genome-Sequencing (WGS)
Abdeckung	250 kb–5 Mb, ca. 300 Gene	30 Mb, ca. 20.000 Gene	3200 Mb, ca. 20.000 Gene, Introns
Kosten/Probe	250–500 €	750 €	Ca. 1500 €
Ø Zahl identifizierter Varianten	Abhängig von der Panel-Größe	Ca. 20.000	Ca. 4.000.000
Möglichkeit der Variantenerkennung	SNVs, Indels, CNVs innerhalb des Panels	SNVs bis 50 bp, Indels	SNVs, Indels, kodierende und nicht-kodierende DNA-Abschnitte, mtDNA, regulatorische Elemente, Repeat-Expansions, strukturelle Varianten, Kopienzahlveränderungen und strukturelle Rearrangements
Flexibilität	+	++	+++
Datenmenge	+	++	+++
Bioinformatik	+	++	+++
Turnaround-Time	+	++	+++
Vorteile	Hohe Kosteneffizienz	Geringe Kosten, flächendeckende Verfügbarkeit, kurze Turnaround-Time	Anpassungsfähigkeit und damit zukunftsfähige Methode, Bewertung struktureller Auffälligkeiten/Tandem-Repeats gegeben, keine Notwendigkeit der Library-Prep
Nachteile	Wenig Flexibilität nach abgeschlossener Gestaltung eines Genpanels	Notwendigkeit der PCR-basierten Library-Prep, fehlende Abdeckung nicht-kodierender DNA, fehlende Möglichkeit der Bewertung struktureller Auffälligkeiten/Tandem- Repeats	Hohe Kosten, große Datenmenge, hohe Wahrscheinlichkeit der Generierung von Varianten unklarer Signifikanz

*bp* Basenpaare, *CNVs* Copy Number Variants, *Indels* Insertionen/Deletionen, *Library-Prep* erster Schritt des NGS, welche die Bindung der DNA-Fragmente an die Flow Cell ermöglicht, *mtDNA* mitochondriale DNA, *SNVs* Single Nucleotide Variants, Einzelbasenaustausch, *Tandem-Repeats* mehrfach wiederholtes Auftreten bestimmter Nukleotidmuster im DNA-Strang, welches teilweise krankheitsassoziiert auftritt, *NGS* Next-Generations Sequencing

## Pilotprojekte für das genomische Neugeborenenscreening

Die Umsetzung eines genomischen Neugeborenenscreenings (gNBS) wird derzeit intensiv diskutiert [21–23]. Als genetische Untersuchungsmethode könnte in einem einzigen Testverfahren eine große Zahl monogener Erkrankungen erfasst werden, welche mit der Methodik des derzeit verwendeten Neugeborenenscreenings nicht erkannt werden können. Mehrere Zentren und Initiativen haben bereits erste Schritte unternommen, um rechtliche, ethische und soziale Rahmenbedingungen eines solchen Screenings zu eruieren, auch technische Aspekte der Machbarkeit eines gNBS wurden bereits untersucht.

Das US-amerikanische *Newborn-Sequencing-in-Genomic-Medicine-and-Public-Health-(NSIGHT-)*Konsortium untersucht in 4 Teilprojekten unterschiedlichste Aspekte eines gNBS [24]. Das BabySeq-Projekt, eine Zusammenarbeit zwischen dem *Brigham and Women’s Hospital*, dem *Boston Children’s Hospital*, dem *Broad Institute*, der *Harvard University* sowie dem *Baylor College of Medicine,* hat zum Ziel, die medizinischen, psychologischen und wirtschaftlichen Auswirkungen der Genomsequenzierung von gesunden und kranken Neugeborenen zu untersuchen [25, 26]. Die Auswahl der 954 zu screenenden Erkrankungen, für die (wahrscheinlich) pathogene Varianten in 889 verschiedenen Genen ursächlich sind, erfolgte auf Grundlage eines mehrstufigen Prozesses [27–29]. Im Rahmen einer ersten Studie wurden 127 gesunde und 32 kritisch kranke Neugeborene mittels WES untersucht. Bei 15/159 (9,4 %) Neugeborenen konnte eine genetische Veränderung identifiziert werden, welche mit dem Auftreten einer behandlungsbedürftigen Erkrankung des Kindesalters assoziiert war. Nach einer Anpassung des Studienprotokolls [30, 31] wurden neben Genveränderungen mit Konsequenz im Kindesalter bei 3/85 (3,5 %) Neugeborenen auch solche Varianten berichtet, die mit einer Erkrankung des Erwachsenenalters assoziiert waren. Hierzu zählten die Anlageträgerschaft für ein Krebs-Prädispositions-Syndrom sowie die Anlageträgerschaft für eine Herzmuskelschwäche. Eine Heterozygotie für autosomal-rezessive Erkrankungen wurde bei insgesamt 88 % der untersuchten Neugeborenen festgestellt [29, 32]. Damit konnte die Studie demonstrieren, dass genetische Veränderungen häufig sind und diese zuverlässig durch ein genomisches Screening identifiziert werden können. Ob hieraus ein klinischer Zusatznutzen für die Versorgung von Betroffenen erzielt werden kann, bleibt bislang offen. In der BabySeq-Studie zeigten Eltern ein hohes Interesse daran, Ergebnisse über genetische Veränderungen zu erhalten, auf deren Grundlage ihr Neugeborenes im Kindesalter (86,8 %) oder im Erwachsenenalter (84,6 %) eine Krankheit entwickeln könnte, die verhindert, behandelt oder geheilt werden kann. Nur etwa die Hälfte der Probanden sprach sich dafür aus, Ergebnisse zu nicht behandelbaren Erkrankungen des Kindes- und Erwachsenenalters zu erhalten [33].

Im Rahmen der *North-Carolina-Newborn-Exome-Sequencing-for-Universal-Screening-*(NC-NEXUS-)Studie wurde eine altersabhängige, semiquantitative Metrik entwickelt, um die klinische Behandlungsfähigkeit von Gen-Erkrankungs-Assoziationen zu bewerten. Von 822 identifizierten Gen-Erkrankungs-Paaren wurden 466 (56,7 %) behandelbare, 245 (29,8 %) nicht oder unzureichend behandelbare Erkrankungen mit Beginn im Kindesalter sowie 25 (3 %) behandelbare und wiederum 19 (2,3 %) nicht oder unzureichend behandelbare Erkrankungen mit Beginn im Erwachsenenalter identifiziert. 67 Erkrankungen (8,2 %) wurden aufgrund unzureichender Datenlage und/oder pränatalen Beginns ausgeschlossen, sodass insgesamt 755 Erkrankungen berichtet wurden [34]. Im direkten Vergleich zur BabySeq-Studie zeigten sich Unterschiede bei der Definition der Gen- und Erkrankungs-Einschlusskriterien, welche letztlich auch in diskrepanten Gen-Listen resultierten. Nachfolgend wurden im Rahmen einer verblindeten, randomisierten Fall-Kontroll-Studie 61 gesunde Neugeborene, 17 Kinder mit einer bereits diagnostizierten neurometabolischen Erkrankung und 28 Kinder mit angeborener Hörschädigung untersucht. 15/17 (88,2 %) Patienten konnten mittels WES analog zu einem auffälligen MS/MS-Screening identifiziert werden. Bei einem Patienten mit autosomal-rezessiver Ahornsirupkrankheit konnte lediglich eine heterozygote, also auf einer der beiden Genkopien vorkommende Missense-Variante im *BCKDHA*-Gen identifiziert werden. Eine zweite kausale Variante wurde nicht gefunden. Im zweiten falsch-negativen Fall wurde bei einem Patienten mit Malonyl-CoA-Decarboxylase-Mangel eine homozygote, also auf beiden Genkopien vorkommende Variante im *MLYCD*-Gen identifiziert, die aufgrund fehlender klinischer Evidenz als Variante unklarer Signifikanz (Klasse-3-Variante) interpretiert wurde. In der Hörverlust-Kohorte konnte bei 18/28 (64 %) Patienten eine die Erkrankung erklärende genetische Veränderung identifiziert werden. Verantwortlich hierfür war laut den Autoren, dass Varianten aufgrund der strengen Kriterien aus dem Datensatz gefiltert wurden. In der Kontrollkohorte fand sich bei 4/106 (3,7 %) Neugeborenen eine behandelbare Erkrankung, sodass die durch das Screening zusätzlich gewonnenen Informationen Einfluss auf die weitere klinische Versorgung des einzelnen Patienten hatten [35]. Die Ergebnisse der NEXUS-Studie machen deutlich, dass ein genomisches Screening vorerst als Ergänzung zum MS/MS-basierten NBS nützlich sein könnte, dieses aber nicht komplett ersetzen kann. Die Ergebnisse zeigten zudem, dass die bioinformatische Verarbeitung der Sequenzdaten entscheidend für die Qualität eines Gen-basierten Screenings ist und eine verbesserte Erkennung und Interpretation von Varianten langfristig den positiv prädiktiven Wert der Methode deutlich steigern könnten.

Der am *Rady Children’s Hospital* von Stephen Kingsmore geleitete Studienarm *BeginNGS™* untersuchte die Methodik des rapid-WGS für ein gNBS. Hierbei wurden in einem iterativen Delphi-Verfahren nachgewiesen wirksame und in Leitlinien formulierte Notfallmaßnahmen identifiziert, die bei 457 genetischen Erkrankungen mit intensivmedizinischer Betreuungsnotwendigkeit erforderlich waren. Diese wurden auf 388 Erkrankungen (in 317 Genen) mit einer kumulativen Inzidenz von ~0,9 % reduziert [36, 37]. Die Ergebnisse des Selektionsprozesses sind frei zugänglich.^1^ Anhand von 454.707 Proben der UK Biobank, einer Langzeit-Biobank-Studie im Vereinigten Königreich, wurde retrospektiv ein rapid-WGS simuliert, wobei eine Spezifität von 99,7 % demonstriert werden konnte. Aus der Analyse von Proben von 2208 kritisch erkrankten Neugeborenen und 2168 elterlichen Proben konnten ein negativ prädiktiver Wert von 99,6 % und eine Sensitivität von 88,8 % erhoben werden [38, 39]. *BeginNGS™ *wurde mittlerweile in ein mehrstufiges Studienprogramm übersetzt und sieht eine Implementierung des rapid-WGS für ein gNBS bis zum Jahr 2027 vor.

Im Vereinigten Königreich wurde 2014 vom *Department of Health & Social Care *die Initiative *Genomics England* gegründet, welche eine vom *National Health Service (NIH)* initiierte Forschungsstudie leitet, um die Vorteile, Herausforderungen und praktischen Aspekte der Sequenzierung und Analyse der Genome von Neugeborenen zu untersuchen. Ein großer Fokus des Projekts liegt auf dem öffentlichen Dialog [40]. Bislang sind nur wenige Informationen zu den technischen Abläufen des Projekts bekannt geworden.

Auf nationaler Ebene wird das vom Bundesministerium für Bildung und Forschung (BMBF) über 3 Jahre geförderte Projekt „NEW_LIVES: Genomic NEWborn screening programs – Legal Implications, Value, Ethics and Society“ der Universitäten Heidelberg und Mannheim ethische, rechtliche, gesellschaftliche und psychologische Aspekte eines genomischen Screenings evaluieren. Folgende Forschungsfragen sollen hierbei adressiert werden: 1) Welche Kriterien sind für die Auswahl genetischer Krankheiten für zukünftige gNBS-Programme in Deutschland zu berücksichtigen? 2) Sollten gNBS-Daten zur späteren Verwendung archiviert oder gelöscht werden? 3) Wie sollte der Informations- und Einwilligungsprozess für gNBS gestaltet werden? 4) Wie sollten ein normativer Rahmen und Best-Practice-Empfehlungen für ein gNBS-Programm in Deutschland aussehen? Ziel ist es, sämtliche Fragen im öffentlichen und wissenschaftlichen Diskurs zu beantworten und so die Meinung und Haltung aller involvierten Interessengruppen abzubilden. Um dies zu erreichen, arbeitet NEW_LIVES eng mit NC NEXUS und den Europäischen Referenznetzwerken für seltene Erkrankungen zusammen [41].

## Herausforderungen bei der Entwicklung eines genomischen Neugeborenenscreenings

Ein auf genetischen Daten beruhendes Neugeborenen-Screening erfordert neben der Sicherstellung der Durchführbarkeit (inklusive ausreichender technischer und klinischer Sensitivität und Spezifität) eine eingehende Beurteilung der rechtlichen Rahmenbedingungen. Das Gendiagnostikgesetz sieht die Möglichkeit von genetischen *Reihenuntersuchungen* vor, wenn die Erkrankung „vermeidbar oder behandelbar ist oder [wenn] der [Erkrankung] vorgebeugt werden kann“ (§ 16 Abs. 1/14 Abs. 1 GenDG). Eine genetische Reihenuntersuchung würde vorrangig in einem öffentlichen Interesse im Sinne einer Vor- und Fürsorgepflicht für die zu testende Person definiert werden und müsste gegen das Selbstbestimmungsrecht sowie dessen Recht auf Nichtwissen abgewogen werden – ein Umstand, der nur dann vorliegt, wenn der Nutzen für die betroffene Person über das reine Wissen um eine Erkrankungswahrscheinlichkeit hinausgeht.

Eine der größten Herausforderungen eines gNBS liegt daher in der Identifizierung von Varianten, die mit keinem oder nur einem geringen Krankheitswert einhergehen. Eine resultierende Überdiagnose und Überbehandlung kann mit Ängsten und Einbußen der Lebensqualität einhergehen, denen bestmöglich vorgebeugt werden sollten. Es bestünde zudem die Möglichkeit, genetische Erkrankungen zu identifizieren, für die derzeit keine Therapieoptionen existieren oder lediglich eine unsichere Therapieindikation vorliegt. Ebenso muss der Umgang mit dem Nachweis heterozygoter Varianten diskutiert werden, die lediglich mit der Anlageträgerschaft einer Erkrankung und damit einer Relevanz für die Reproduktion und die Folgegeneration (oder die weitere Familienplanung der Eltern) ohne unmittelbare Auswirkung auf die Gesundheit des Getesteten einhergeht. Die mit der Unsicherheit genetischer Befunde verbundenen Risiken, wie beispielsweise sozialrechtliche Diskriminierung und auch psychologische Folgen für die Eltern-Kind- und die Eltern-Arzt-Beziehung, müssen beachtet werden und sollten bereits im Rahmen einer transparenten Aufklärung thematisiert werden. Eine Überprüfung und ggf. Anpassung der gesetzlichen Grundlagen sind zudem zwingend erforderlich und stellen eine Weichenposition für die Planung und Durchführung von nationalen Implementierungsstudien eines gNBS dar.

**Figure 2:**
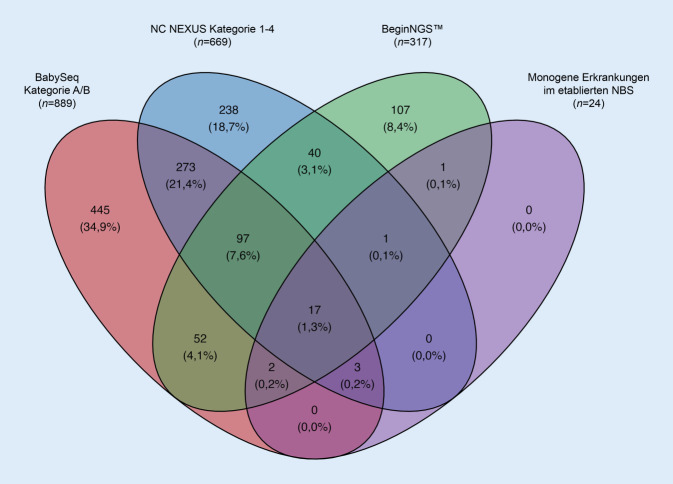

Die Frage, welche *Zielkrankheiten* in einem gNBS untersucht werden sollen, bleibt Gegenstand intensiver Diskussionen. Durch Wilson und Jungner wurden 1968 Kriterien benannt, welche das Fundament für den Diskurs über Nutzen, Schaden, ethische Aspekte und Kosten von Screening-Programmen bis heute begleiten [37]. Was die bislang veröffentlichten Studien vereint, ist der Ruf nach Transparenz bei der Auswahl von Zielkrankheiten und der Bewertung genetischer Veränderungen. Mehrere Systematiken wurden vorgeschlagen (Tab. 2) und bislang publizierte Studien folgten ähnlichen, wenn auch nicht deckungsgleichen Bewertungsprinzipien. In Abb. 2 ist die Überlappung von Zielgenen aus den 3 bislang erschienenen Studien (BabySeq, NC NEXUS und BeginNGS^TM^) und dem derzeitigen Neugeborenenscreening in Deutschland (entsprechend den aktuellen monogen bedingten Zielkrankheiten) dargestellt.

**Table 2:** 

Studie/ Initiative	Kriterien zur Identifikation von Zielerkrankungen für das gNBS	Anzahl Erkrankungen (Gene) in Kategorien
BabySeq [29]	Es gibt eine klare *Gen-Krankheits-Assoziation*. Die Kuration erfolgt durch die Clinical Genome Resource (ClinGen) Gene Curation Working Group, durch die die Validität einer Gen-Krankheits-Assoziation durch Überprüfung der Evidenz in der Literatur bewertet wird. Hierbei findet die Anzahl der betroffenen Familien mit pathogenen Varianten in einem Gen und die Verfügbarkeit von funktionellen Studien Beachtung	**BabySeq-Kategorien**^a^ Kategorie A: 884 (839) Kategorie B: 70 (64) Kategorie C: 560 (542) *Summe: 1514 (1445)*
	*Alter* bei Auftreten erster Symptome: Das Alter, in dem bei Betroffenen mit pathogenen Varianten in einem Gen Symptome der Krankheit erstmals auftraten, eingeteilt in 4 Gruppen: ≤ 2 Jahre, 2–10 Jahre, 10–18 Jahre und > 18 Jahre	
	Die Penetranz der Erkrankung wird als „hoch“ eingestuft, wenn ≥ 80 % der Betroffenen symptomatisch waren, „mäßig“, wenn 20–80 % der Personen symptomatisch waren, und „niedrig“, wenn < 20 % der Personen symptomatisch waren	
	Das am häufigsten berichtete *Vererbungsmuster* für ein Gen	
NC NEXUS [34]	Der *natürliche Verlauf der Erkrankung* ist bekannt	**NC-Nexus-Kategorien**^b^: Kategorie 1: 466 (430) Kategorie 2: 245 (238) Kategorie 3: 25 (25) Kategorie 4: 19 (19) Kategorie 5: 67 *Summe: 755 (712)*
	Die Erkrankung stellt ein *erhebliches Risiko* hinsichtlich Morbidität und Mortalität bei Säuglingen oder Kleinkindern dar	
	Es gibt eine *wirksame Behandlung*, die allgemein akzeptiert wird	
	Eine frühzeitige Behandlung *beeinflusst den Krankheitsverlauf positiv*	
	Die *Vorteile eines frühen Therapiebeginns* überwiegen eindeutig die damit assoziierten Risiken	
	Bei Genen mit mehr als einer assoziierten Erkrankung existieren unterschiedliche therapeutische Optionen. *Unterschiedliche Phänotypen* können zuverlässig durch die genetische Untersuchung unterschieden werden	
BeginNGS™ [39]	**Ist der *natürliche Verlauf* dieser genetischen Krankheit gut bekannt?**	**BeginNGS™-Kategorien**^c^ Kategorie A: 295 (248) Kategorie B: 93 (79) *Summe: 388 (327)*
	Gibt es mindestens eine gut belegte Gen-Phänotyp-Assoziation?	
	Gibt es eine signifikante Variation in der Expressivität?	
	Liegt eine reduzierte Penetranz vor?	
	Ist der Erbgang (autosomal dominant, autosomal rezessiv, X‑chromosomal, mitochondrial) gut verstanden?	
	Ist die Pathogenität (zumindest einer Teilmenge) der beschriebenen DNA-Varianten gut bekannt (Loss-of-Function oder Gain-of-Function)?	
	Ist die Genotyp-Phänotyp-Korrelation für diese Varianten ausreichend, um den Krankheitsverlauf vorherzusagen?	
	Kann die Variabilität des Ergebnisses oder der Schwere der Erkrankung durch zusätzliche Untersuchungen (wie z. B. biochemische, enzymatische Methoden oder einen Funktionstest) geklärt werden?	
	**Stellt diese genetische Erkrankung ein *signifikantes Risiko für Morbidität und Mortalität *bei Säuglingen oder Kleinkindern dar?**	
	Ist die Penetranz hoch genug, sodass die Identifizierung einer klinisch unbedeutenden Krankheit minimal ist oder nur minimalen Schaden verursacht?	
	**Gibt es eine *Behandlung* oder Intervention, die wirksam und akzeptiert ist?**	
	Steht eine Behandlung zur Verfügung, die den Krankheitsverlauf beeinflussen kann?	
	Ist die Behandlung für alle betroffenen Personen wirksam?	
	Ist das Ansprechen auf die Behandlung bei bestimmten pathogenen Variante(n) einheitlich?	
	Ist die Behandlung für alle Symptome einer Erkrankung wirksam?	
	Wenn keine spezifische Behandlung verfügbar ist, würde eine Diagnose die Behandlung auf andere Weise verändern?	
	Ist eine Behandlung allgemein verfügbar und gibt es genügend Anbieter, Einrichtungen und Ressourcen, um alle identifizierten Personen zu versorgen?	
	Ist eine Behandlung für die Mehrheit der Bevölkerung akzeptabel? Dabei sind die Kosten, die Morbidität der Behandlung sowie religiöse oder politische Überzeugungen zu berücksichtigen. Erfordert dieser Eingriff beispielsweise die Verwendung von fetalem Gewebe?	
	**Verbessert eine *frühzeitige Behandlung* das Ergebnis?**	
	Gibt es eine Latenzphase, in welcher der Beginn der Behandlung zu einem besseren Ergebnis führt oder Komplikationen verhindert?	
	Führt eine verzögerte Diagnose zu einem schlechteren Langzeitverlauf oder zu schweren Komplikationen?	
	Führt eine frühzeitige Diagnose und Behandlung zu einem besseren Langzeitverlauf als ein Behandlungsbeginn nach Auftreten der Symptome?	
	**Überwiegen die *Vorteile eines frühzeitigen Eingreifens* eindeutig die Risiken?**	
	Sind falsch-positive Ergebnisse bei diesem Gen problematisch?	
	Könnte die Einführung des gNBS bei dieser Erkrankung einen negativen Nettonutzen haben? Die Überlegungen beziehen sich auf den Probanden, die Familie und die Allgemeinbevölkerung	
	Bestehen Bedenken hinsichtlich der Identifizierung von heterozygoten Anlageträgern?	
	**Gibt es bei *Genen mit mehr als einer assoziierten Erkrankung* Unterschiede in der Behandlung, und können sie durch rWGS oder zusätzliche Tests unterschieden werden?**	
Genomics England [40]	Es gibt überzeugende Beweise dafür, dass die *genetische(n) Variante(n) die Krankheit verursacht/verursachen und zuverlässig nachgewiesen* werden kann/können	Bislang keine Erkrankungs‑/Genliste verfügbar
	Es ist davon auszugehen, dass *ein hoher Anteil der Personen, die die genetische(n) Variante(n) aufweisen, Symptome haben*, welche die Lebensqualität beeinträchtigen würden, wenn sie nicht diagnostiziert würden	
	Eine *frühzeitige oder präsymptomatische Behandlung der Erkrankung* führt bei Kindern nachweislich zu wesentlich *besseren Ergebnissen als eine Behandlung* nach Auftreten der Symptome	
	Bei den untersuchten Erkrankungen handelt es sich nur um solche, bei denen die Interventionen *für alle gleichermaßen zugänglich* sind	

^a^ BabySeq-Definition der *Kategorie A* in das gNBS aufgenommene Gene mit definitiven oder starken Hinweisen darauf, dass sie eine hochpenetrante, bei Kindern auftretende Störung verursachen; *Kategorie B* Gene, die in das gNBS aufgenommen wurden, basierend auf einer Handlungsfähigkeit im Kindesalter mit mäßiger Evidenz oder mäßiger Penetranz, für die fachliche Leitlinien oder Expertenmeinungen festgestellt haben, dass nicht-invasive Eingriffe den Verlauf der Erkrankung wahrscheinlich verbessern können; *Kategorie C* Gene, welche die Kriterien für die Aufnahme in ein gNBS *nicht* erfüllen. In der BabySeq-Studie wurden nur (wahrscheinlich) pathogene Varianten aus Kategorie A und B berichtet

^b^ NC-NEXUS-Definition der *Kategorie 1* Erkrankungen mit Beginn im Kindesalter und guter Evidenzlage für verfügbare Therapien; *Kategorie 2* Erkrankungen mit Beginn in der Kindheit und geringer bis keiner Evidenzlage für verfügbare Therapien; *Kategorie 3* Erkrankungen mit Beginn im Erwachsenenalter und guter Evidenzlage für verfügbare Therapien; *Kategorie 4* Erkrankungen mit Beginn im Erwachsenenalter und geringer bis keiner Evidenzlage für verfügbare Therapien; *Kategorie 5* kontroverse Erkenntnisse und/oder pränataler Beginn. In der NC-NEXUS-Studie wurden nur (wahrscheinlich) pathogene Varianten aus Kategorie 1 bis 4 berichtet

^c^ BeginNGS™-Definition der *Kategorie 1* Erkrankungen, für die es keine größeren Evidenzlücken, eine hohe Nutzenwahrscheinlichkeit und ein geringes Schadensrisiko gab; *Kategorie 2* Erkrankungen, für die es Lücken in der Evidenz oder Unsicherheiten hinsichtlich des Nettonutzens gab, die eine weitere Bewertung erforderten

Für das Screening einer Erkrankung werden eine hohe Evidenz der Kausalität genetischer Veränderung/en für die daraus resultierende Erkrankung sowie hohe Penetranz gefordert und die Existenz einer für alle Menschen gleichermaßen zugänglichen und wirksamen Behandlung vorausgesetzt. Gerade was die Wirksamkeit therapeutischer Interventionen angeht, fehlt für viele genetische Erkrankungen bislang verwertbare Evidenz. Internationale Register für die strukturierte Erfassung und Langzeitstudie von Betroffenen müssten hierfür ausgebaut und intensiv genutzt werden, insbesondere für Patienten mit seltenen Erkrankungen.

Bei ganzgenomischen Analysen besteht die Möglichkeit, dass *Zufallsbefunde* generiert werden, die *per definitionem* nicht im Zusammenhang mit der direkten Indikation der angeforderten Untersuchung stehen und damit über die Aufgabe eines klassischen NGS hinausgehen. Dies beinhaltet im Falle der Untersuchung Minderjähriger beispielsweise genetische Varianten, welche zu Erkrankungen im Erwachsenenalter führen, oder aber Veränderungen in Genen, die mit einem Tumor-Prädispositions-Syndrom einhergehen und damit zum Zeitpunkt der Ermittlung für den Getesteten nur mittelbar relevant sind, für weitere Familienmitglieder (wie bspw. die Eltern) jedoch unmittelbare medizinisch-therapeutische Konsequenzen, wie den Einschluss in Vorsorgeprogramme, haben könnten. Der Umgang mit Zufallsbefunden ist in der Richtlinie der Gendiagnostik-Kommission zur Aufklärung bei genetischen Untersuchungen^2^ geregelt. Er muss im Vorfeld sowohl durch eine weitere Klärung der gesetzlichen Rahmenbedingungen und durch Leitlinien der offiziellen Gremien in Bezug auf ein gNBS definiert werden als auch den Teilnehmenden in einem transparenten Aufklärungsgespräch dargelegt werden. Es sollten zudem klinische Daten über den Nutzen und Schaden der Rückmeldung von Ergebnissen gesammelt werden, um zukünftige politische Diskussionen zu unterstützen.

Unklar bleibt zudem, wie mit Genen umgegangen werden soll, für die eine inkomplette *Penetranz* beschrieben ist, bei denen ein Vorliegen einer (auch pathogenen) genetischen Veränderung nicht immer zur Merkmalsausprägung bzw. zum Ausbruch der Erkrankung führt. Einen Lösungsansatz hierfür bieten strukturierte Bewertungskriterien für die Erstellung von Gen-Erkrankungs-Paaren unter Berücksichtigung der Penetranz anhand der aktuell verfügbaren Literatur.

Veränderungen in unterschiedlichen Genen können außerdem überlappende oder identische Erkrankungen verursachen (*Heterogenität*). Ebenso können Veränderungen in einem einzelnen Gen zu unterschiedlichen Phänotypen führen (*Pleiotropie*). Dieser Umstand sollte in die Bewertung genetischer Veränderungen eines gNBS-Ansatzes einfließen, um eine an die Lebensumstände des Betroffenen adaptierte Informationsnutzung zu ermöglichen.

## Chancen eines genomischen Neugeborenenscreenings

**Figure 3:**
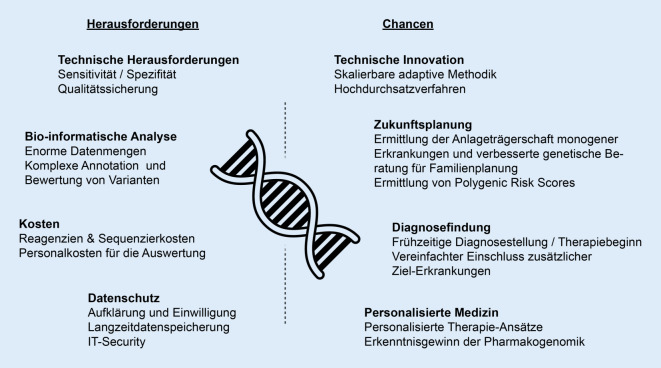

In ersten Pilotstudien konnte gezeigt werden, dass die Genomsequenzierung letal verlaufende Erkrankungen identifizierbar macht und Betroffenen und deren Familien Informationen bezüglich einer genetischen Prädisposition für später im Leben auftretende Erkrankungen liefern kann [29, 31, 35]. Abb. 3 zeigt eine Übersicht der Chancen und Herausforderungen eines gNBS.

Das ACMG stellt regelmäßig Orientierungshilfen für die Meldung von *Zufallsbefunden* im Zusammenhang mit der klinischen Exom- und Genomsequenzierung zur Verfügung [42, 43]. Die aktuelle Version (v3.1) beinhaltet 78 Gene, für die bei Vorliegen pathogener und wahrscheinlich pathogener Varianten eine Befundmitteilung an die getestete Person aufgrund einer klinisch-therapeutischen Relevanz empfohlen wird. Eine Überlappung der Gene, die sich in der ACMG-Liste finden, und einer zukünftigen Liste von Genen, die in einem gNBS untersucht werden sollen, ist durchaus denkbar. Eine unbeabsichtigte Identifizierung von (wahrscheinlich) pathogenen Varianten in Genen der ACMG-Liste – die jedoch nicht im gNBS untersucht werden sollen – könnte dennoch einen über die getestete Person hinausreichenden informativen Wert für weitere Familienangehörige haben, da humangenetische Beratung, individuelle Testung und/oder der Einschluss in strukturierte Früherkennungs- und Vorsorgeprogramme sowie die Inanspruchnahme prophylaktischer therapeutischer Interventionen ermöglicht werden. Auch für die weitere Familienplanung könnten Erkenntnisse, die als Zusatzbefund gewertet werden, entscheidenden Einfluss haben. Ein großer Vorteil gegenüber bisheriger Screening-Methoden ist die fehlende Notwendigkeit biochemischer Marker und eine damit einhergehende deutliche Verkürzung des Zeitraums, bis eine Erkrankung mit eindeutiger genetischer Ursache dem Screening-Panel hinzugefügt werden kann.

Ein verbessertes Verständnis der genetischen Grundlagen von Krankheiten verspricht zu besseren und vor allem zu zielgerichteteren Therapieoptionen zu führen. Sogenannte Targeted Therapies stehen hierbei für den Einsatz von Therapeutika, deren Auswahl sich am genetischen Hintergrund des Betroffenen orientiert. Der Einsatz von WES/WGS für die verbesserte pharmakologische Therapie von Betroffenen wurde bereits vielfach und in Bezug auf unterschiedlichste Erkrankungen demonstriert [44–46].

Zusätzlich zum direkten Nutzen eines gNBS für das Neugeborene wird durch die Generierung großer genomischer Datenmengen auch das Verständnis genetischer Variationen verbessert und kann so nachhaltig die Entscheidungsfindung im Rahmen der Präzisionsmedizin („precision medicine“) beeinflussen und verbessern. Grundlegend hierfür sind nationale Strukturen, in denen genomische Daten systematisch gespeichert und ausgewertet werden können. Die nationale Initiative „genomDE“ wurde ins Leben gerufen, um die Genomsequenzierung von Patienten in der Regelversorgung zu ermöglichen. Hierfür sollen sichere Strukturen für den Transfer und die Langzeitspeicherung von genomischen Daten auf nationaler Ebene errichtet werden, um langfristig verschiedene Einrichtungen der klinischen Versorgung unter Einhaltung ethischer, regulatorischer und rechtlicher Aspekte zu verbinden. „genomDE“ wurde 2020 an die europäische Initiative „1+Million Genomes“ angegliedert, welche zum Ziel hat, über eine Million Genome zu sequenzieren, um damit die Gesundheitsversorgung nachhaltig zu verbessern [47]. Zentrale Punkte der politischen Diskussion sind die koordinierte Nutzung genomischer Daten für die Forschung und deren Transfer in die klinische Versorgung. Nationale gNBS-Projekte könnten daher in größer angelegte (inter)nationale genomische Initiativen eingebunden werden und so entstehende Datenbankstrukturen mitgestalten, um die langfristige Nutzung und nachfolgende Nutzenbewertung zu erleichtern.

## Fazit

Die Anwendung von Hochdurchsatz-Sequenziermethoden für die Durchführung genetischer Reihenuntersuchungen als Neugeborenenscreening bietet große Chancen für die Verbesserung der Bevölkerungsgesundheit. Aufgrund von genetischer Heterogenität, Herausforderungen bei der Identifizierung und Interpretation von genetischen Varianten sowie dem daraus resultierenden Umgang mit unsicherer Information wird ein gNBS aktuell gängige Screening-Methoden nicht vollständig ersetzen können. Der entscheidende Vorteil eines WES/WGS-basierten Screenings wäre jedoch eine an ständig aktualisierte Datengrundlagen adaptierte und damit skalierbare Auswahl der zu screenenden Erkrankungen und eine Methodik, welche die Diagnose nahezu jeder genetischen Erkrankung ermöglichen würde. Damit scheint ein genombasierter Ansatz beim Neugeborenenscreening die logische Konsequenz der technischen Innovation im Feld der Humangenetik zu sein. Es ist nur eine Frage der Zeit, bis die technischen Herausforderungen bewältigt und offene Fragen der Varianteninterpretation beantwortet sind und damit die Sensitivität und Spezifität der Methodik für ein populationsbasiertes Screening ausreichen. Angepasste gesetzliche Rahmenbedingungen und ein transparentes Einwilligungsverfahren vorausgesetzt, könnte ein gNBS unsere Art der Früherkennung potenzieller Erkrankungen und genetischer Erkrankungsdisposition grundsätzlich verändern. Ein deutlicher Rückgang der mit DNA-Sequenzierung und Varianten-Interpretation assoziierten Kosten lässt die finanzielle Machbarkeit genetischer Sekundärprävention im klinischen Alltag zunehmend greifbar erscheinen. Langfristig wird aus unserer Sicht der persönliche, gesundheitswirtschaftliche, aber auch der gesellschaftliche Nutzen eines gNBS überwiegen, jedoch liegt vor uns noch ein weiter Weg. Der öffentliche Diskurs über die Möglichkeiten und Grenzen eines gNBS stellt hierfür ein zentrales Mittel dar, um Verständnis und Akzeptanz eines gNBS in der Bevölkerung zu erreichen und so den Nutzen für die Gemeinschaft zu maximieren.

## Footnotes

1 Rady Children’s Institute for Genomic Medicine Realm: https://gtrx.radygenomiclab.com/about.html; Zugriff: 12.09.2023.

2 Richtlinie der Gendiagnostik-Kommission (GEKO) für die Anforderungen an die Inhalte der Aufklärung bei genetischen Untersuchungen zu medizinischen Zwecken gemäß § 23 Abs. 2 Nr. 3 GenDG.
